# Ceramic Materials and Technologies Applied to Digital Works in Implant-Supported Restorative Dentistry

**DOI:** 10.3390/ma13081964

**Published:** 2020-04-22

**Authors:** Se-Wook Pyo, Dae-Joon Kim, Jung-Suk Han, In-Sung Luke Yeo

**Affiliations:** 1 Department of Dentistry, Ajou University School of Medicine, Suwon 16499, Gyeonggi-do, Korea; 2 Acucera Inc., Pocheon 11192, Gyeonggi-do, Korea; 3 Department of Prosthodontics, School of Dentistry and Dental Research Institute, Seoul National University, Seoul 03080, Korea

**Keywords:** computer-aided design, analog-digital conversion, yttria-stabilized tetragonal zirconia, lithium disilicate, implant-supported dental prosthesis

## Abstract

Computer-aided design and manufacturing technology has been closely associated with implant-supported restoration. The digital system employed for prosthodontic restorations comprises data acquisition, processing, and manufacturing using subtractive or additive methods. As digital implantology has developed, optical scanning, computer-based digital algorithms, fabricating techniques, and numerical control skills have all rapidly improved in terms of their accuracy, which has resulted in the development of new ceramic materials with advanced esthetics and durability for clinical application. This study reviews the application of digital technology in implant-supported dental restoration and explores two globally utilized ceramic restorative materials: Yttria-stabilized tetragonal zirconia polycrystalline and lithium disilicate glass ceramics.

## 1. Introduction

A close association between computer-aided design (CAD)/computer-aided manufacturing (CAM) technology and implant dentistry has been developed since 1973, when Duret first performed dental restorative treatment using digital skills [1]. The CAD phase consists of data acquisition, design for provisional or definitive restoration, and implant installation planning based on the restorative design in a virtual space or using software. A subtractive milling machine cuts a block of restorative material to make the CAD product during the CAM phase, using cutting tools and the calculated paths of these tools. Recently, additive manufacturing technology has been introduced in various clinical dental fields and used to produce implant-supported restoration in digital works [2].

Some dental CAD/CAM systems are used to make prosthodontic restorations in clinicians’ offices (in-office type), which are delivered to patients’ mouths on the day of treatment [3]. Many dental CAD/CAM systems produce their products in dental laboratories (in-lab type). The in-office type of CAD/CAM system has a narrow range of applications limited to inlays, porcelain laminate veneers, and single crowns because of the conditions in the clinic [4,5]. However, as the in-office type is an all-in-one system containing data acquisition, processing, and manufacturing, many restorations have been made with this in-office CAD/CAM and used clinically [5,6]. The in-lab type produces not only single restorative modalities, but also long-span fixed dental prostheses replacing four, five, or even more missing teeth. Such a CAD/CAM system requires transfer of the oral data of a patient to the dental laboratory. A stone model is scanned using an extraoral scanner, while the digital impression technique with an intraoral scanner is employed for conventional impression-taking step in the clinic. Using the intraoral scanner, dental clinicians can reduce the time required to initiate the design process for restorations and electronically send the acquired data to any dental laboratory in the world [7].

Subtractive or additive manufacturing is used to process materials, including metals, ceramics, and polymers, for implant-supported restorations [2,8]. The materials required for different CAD/CAM systems have been developed and applied in various ways, depending on the type of processing equipment involved. Subtractive manufacturing (SM), mainly the milling method, is used for directly processing ceramic or metal ingots for definitive prostheses. In the field of implant-supported fixed prosthodontics, the ceramic materials have been actively utilized for SM, as excess material waste and esthetics limit the use of metals (including gold) [2]. SM is also applied to polymers like polymethylmethacrylate for provisional restorations. Generally, SM produces dental prostheses accurately, and this technology has been well established in dentistry. A disadvantage is that SM leaves much waste because of a significant amount of loss of a material from milling the material block [2,8]. Another important limitation is that the degrees of freedom of the equipment, including the number of milling axes and the dimension of tools, affect the reproduction of an object, especially when the object is morphologically complex [8]. Additive manufacturing (AM) is advantageous in material waste and the reproducibility of a complex body. AM also reduces the residual stress from tooling, which frequently occurs in SM [2]. However, the dimensional accuracy, surface-finishing quality and mechanical properties of an object from AM are still under investigation in the dental field [2,8]. AM utilizes plastic or wax materials, producing burnout patterns that are subsequently changed to metal restorations by casting, or to all-ceramic restorations by the heat-pressing of lithium disilicate glass. Recently, large-scale metal frameworks for fixed implant-supported prostheses and removable partial dentures have been directly printed three-dimensionally, using cobalt-chromium or titanium alloys and a technology that selectively melts metal powder with lasers (selective laser melting) [9,10,11]. However, the clinical application of AM has not yet been realized for ceramic restorations.

## 2. Data Acquisition, Processing, and Manufacturing in Digital Workflow

**Figure 1:**
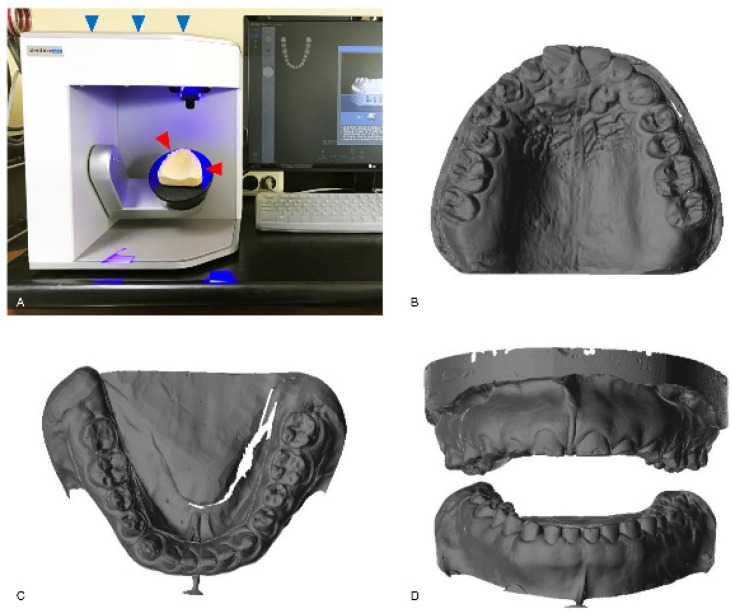
The indirect method for data acquisition. A conventional gypsum model (red arrowheads) is scanned with a model scanner (blue arrowheads; Identica hybrid, Medit, Seoul, Korea) (**A**). The scanned image data of the maxilla (**B**) and the mandible (**C**) are shown. The frontal view of the scanned data for both arches is also shown (**D**).

**Figure 2:**
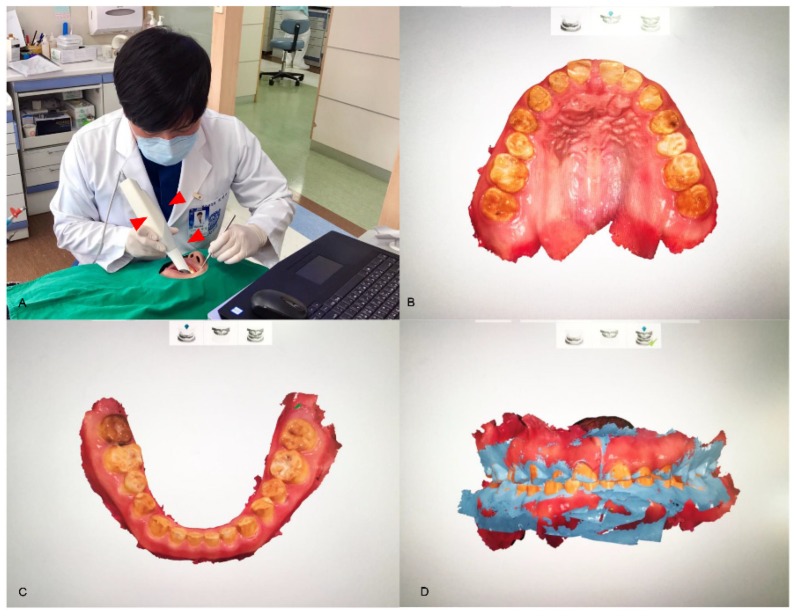
The direct method for data acquisition. An intraoral scanner (red arrowheads; TRIOS, 3Shape, Copenhagen, Denmark) converts the analog information of a patient’s mouth into digital data (**A**). The directly scanned data of the maxilla (**B**) and the mandible (**C**) are shown. The articulations between the arches are able to be digitally handled, depending on the jaw relation of the patient (**D**).

There are several ways to obtain the first set of digital data in the workflow that is to be processed for CAD. The image data are acquired either indirectly or directly. The indirect method comprises the traditional analog procedure and the conversion of the analog information into digital data in the clinical process. After taking an impression of the teeth or whole dentition using an impression material, like irreversible hydrocolloid or polyvinylsiloxane, the impression itself or the negative form of the information is scanned three-dimensionally, creating digital data for further CAD processing. Instead of the impression, a dental model is produced in the conventional manner, such as by pouring gypsum into the impression, and this model is used for scanning, whereby a dental model scanner converts the information into digital data (Figure 1). The direct method is completely digitalized, and data on the patient’s mouth is obtained using an intraoral scanner (Figure 2). When acquiring data with the intraoral scanner, the image information from the scanner is directly stored in the computer hardware. The acquired image data can be utilized to make a model for prosthodontic restorations. Simple restorations, such as single crowns, are sometimes fabricated from the image data without the model, which is called the model-less approach [12]. However, a working model is usually produced using SM or AM for more precise clinical treatment of a patient, especially when porcelain firing or ceramic sintering is required for the coping process and when the model is necessary for harmonization of the prosthesis with the adjacent and antagonistic teeth [13].

**Figure 3:**
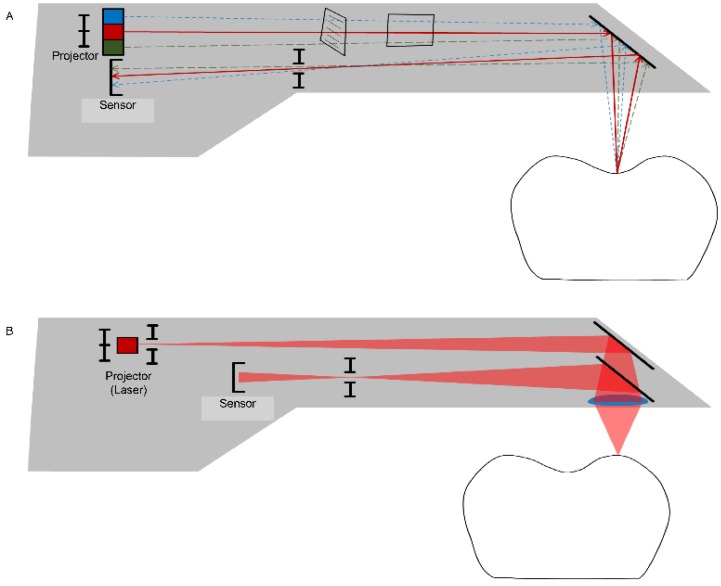
Data-capturing principles for an intraoral scanner use a variety of techniques, such as active triangulation (**A**) and confocal (laser) microscopy (**B**) to create a focus distance within the limited space of the probe.

Both methods (direct and indirect) employed for data acquisition are based on the technology of optical scanning. The accuracy of an extraoral scanner is accepted as satisfactory for the clinical level, while many efforts are being made to develop an intraoral scanner that has an accuracy similar to that of an extraoral scanner [14]. Because an intraoral scanner should acquire the necessary data when capturing images of small oral structures that have many undercuts in a limited space, the data-capturing principles of the device are different from those of the extraoral scanner, which generally has a camera fixed at the top. The CEREC system (Dentsply Sirona Inc., York, PA, USA) initially used the active triangulation principle (Figure 3A) and has recently added video sequence technology in data-capturing [14,15,16]. The iTero (Align Technology, Inc., San Jose, CA, USA) and TRIOS (3Shape, Copenhagen, Denmark) intraoral scanners are based on the principles of confocal microscopy (Figure 3B) [15,16]. The focal depth or recognition range of intraoral scanners depends on the data capture principles [17]. With confocal microscopy intraoral scanners, for example, a scan wand must be turned to the buccal or lingual side to scan deeper areas, particularly when the focal depth is shallow, the abutment teeth are long, or the gap between adjacent teeth is narrow. If the scanning software that overlaps and recombines the acquired data has poor performance, obtaining an accurate 3D model is difficult, and possibly involves a longer scan time [17]. Powder coatings on oral structures represent another issue. Some intraoral scanner systems require that powder be applied to the tooth surface before scanning for clearer data acquisition [17]. Powder is used to increase the recognition rate of scanners by highlighting objects with different surface reflectance values, such as enamel and porcelain, and by reducing the reflectivity. However, the use of powder remains controversial due to the associated dangers of powder being placed in oral cavities and difficulties in uniformly spraying the powder on oral structures. The powder also needs to be washed away, especially from the tongue and buccal cheek, and can be easily contaminated by the saliva in patients’ mouths [18,19]. To address this issue, most intraoral scanners have evolved in recent years such that scanning can be performed without the requirement for powder spraying [17,20]. Currently, intraoral scanners have been reported to be as accurate as extraoral scanners for implant-supported restorations [21,22,23]; however, these studies were only performed in vitro. A previous human study showed that for implants inserted into patients’ jaws, the distance and angulation errors of intraoral scanning were large, and concluded that it is difficult to make well-fitting implant-supported restorations for completely edentulous mandibles when the data of the inserted implants have been acquired with intraoral scanners [24].

When digitized data are obtained from scanners, these data are loaded into dental CAD software to design implant-supported prostheses and frameworks, which are realized through the CAM process. It is unnecessary to produce a model from the data in the case of an extraoral scanner, as the data are acquired from the dental cast obtained from conventional impression-taking. The digitized data often need to be transformed into a real three-dimensional model when the information of the oral structures is digitized by an intraoral scanner. For a simple case, like a single implant-supported crown, a physical model is not fabricated, which is called model-less production. For an extensive case including full mouth rehabilitation, the data are printed or milled into a real three-dimensional model to define the proximal relationship between the adjacent and opposing teeth for the porcelain build-up procedure, which should be manually performed to achieve the final shape.

Intraoral scan data are used for both model manufacturing and restoration design [25]. The working model is manufactured by SM (for example, computer numerical control (CNC) milling) or AM (for example, rapid prototyping). For the milling process, polyurethane or polymethyl-methacrylate resin blocks with abrasion resistance are used for laboratory work, and the corresponding resin is used for three-dimensional printing through rapid prototyping [26,27]. The design of the working model is completed using dental CAD software, which is based on optical impression data collected by an intraoral scanner. The movable tissue area unnecessary for prosthodontic restorations is cut to optimize the total volume of the data. Articulation—or the occlusions between the upper and lower arches, which are automatically aligned by the buccal vestibular bite—is checked and detailed corrections are made if necessary. The clinician should review the optical impression immediately after scanning and ensure that all critical parts, including the margins required for the restoration, have been captured.

Although it is possible for most dental restorative procedures to be converted into digital works, some limitations still remain. Digital manufacturing systems (both SM and AM) are limited in terms of expressing the subtle surface texture and different hue characteristics of natural teeth, which are critical for the esthetic restoration of anterior teeth. Manual handling by experienced technicians is required to reproduce these characteristics. The type of data acquired by a digital scanner is a point, which is stored as point cloud data. The data are processed to the form of a triangle or polygon, which has the minimum unit of a plane. Therefore, it is impossible to recognize borderline conditions using digitized data, so these have to be approximated [28,29]. For this reason, it is extremely difficult to delicately reproduce the emergence profile of prosthodontic restorations at the margin without manual adjustment.

## 3. Materials Used in Data Manufacturing for Implant-Supported Restoration

Dental materials used by digital technology have been developed with the advancement of dental CAD/CAM equipment. SM has been widely used to fabricate tooth-supported ceramic restorations for over 30 years and implant-supported ceramic restorations for over approximately 20 years [30,31]. SM has been unavailable for metal (including gold) restorations due to esthetics and excess material waste [2]. Polymers are the most frequently used materials in AM for dental applications [32]. However, these materials are only employed for provisional restorations and not for definitive restorations. AM has not yet been clinically applied to ceramic dental restorations, mainly due to the difficulties in producing definitive prostheses with suitable surface finishing, mechanical properties, and dimensional accuracy [8].

**Figure 4:**
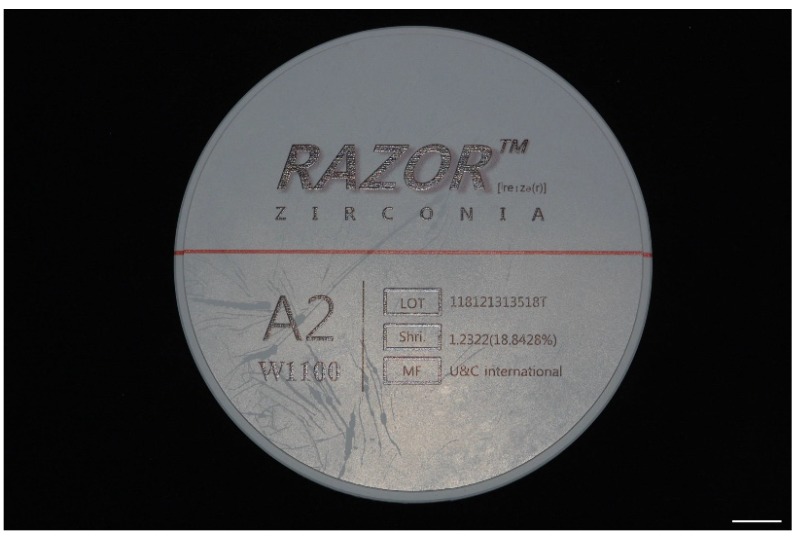
A disc-shaped zirconia block (Razor, U&C International Co., Ltd., Seoul, Korea). This block is partially sintered and 98 mm in diameter, and is available for a full arch implant-supported restoration. Bar = 1 cm.

**Figure 5:**
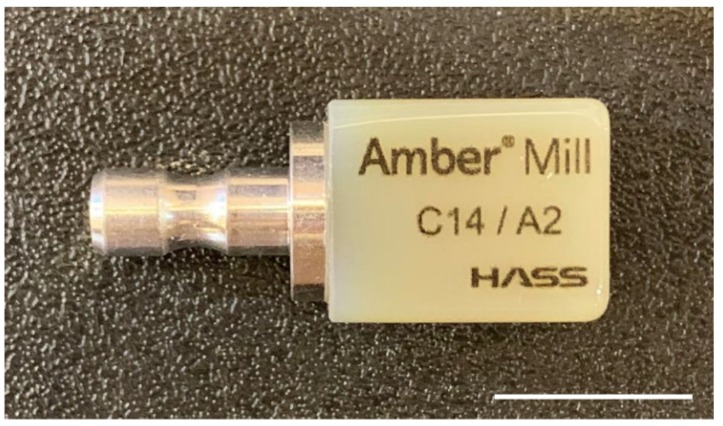
A bar-shaped lithium disilicate block (Amber Mill, HASS Co., Gangneung, Korea). This rectangular block is 14 mm in height, and is available for single restorations. Bar = 1 cm.

Disc-shaped materials are used for SM in dental laboratories or milling centers (Figure 4). Milling machines are categorized as dry or wet, depending on whether a coolant is irrigated or not. Zirconia blocks processed by both types of milling machine should be dried before coloring and sintering. Therefore, dry machining is widely applied in the processing of zirconia, especially for partially sintered blanks, because of its weakness in water [33]. Other ceramics, such as feldspathic porcelain and lithium disilicate glass, zirconia-resin composite materials, and metals, are usually processed with lubricants during milling. Soft metals are ground using a carbide bur, whereas strong metals, ceramics, and hard resins are ground using an electroplated diamond bur. Therefore, appropriate equipment should be selected in order to meet the requirements of the processed materials. The SM machines that are used for restorations aimed at the same day delivery in the dental clinic are driven in a unique manner, wherein the burs are approached from both the left and right sides to shorten the machining time. A bar-shaped block material is used for these cases (Figure 5). The inlay, which is the most commonly produced on this platform, is fabricated using a zirconia–resin composite material (Lava Ultimate, 3M Company, Maplewood, MN, USA) [34,35]. A lithium disilicate glass ceramic (IPS e.max CAD, Ivoclar Vivadent Ltd., Schaan, Liechtenstein) has recently been introduced for single implant-supported restoration [36]. A polymer material blank for provisional restoration (VITA CAD-Temp, VITA Zahnfabrik H. Rauter GmbH & Co. KG, Bad Säckingen, Germany) and a zirconia blank (IPS e.max ZirCAD, Ivoclar Vivadent Ltd., Schaan, Liechtenstein) have also been used in the in-office type system for larger prosthodontic restorations, such as 3-unit fixed dental prostheses. In addition, other materials, including Co–Cr alloys and ceramic–polymer composites, are being introduced for in-office use [37].

The major ceramic materials for implant-supported restoration can be classified into two categories: zirconia-based ceramics and glass ceramics.

### 3.1. Zirconia

Zirconia has exceptional mechanical properties that make it resistant to repeated masticatory force and an acceptable machinability at the pre-sintering stage of CAD/CAM [38]. Due to this machinability, CNC milling has been applied, without complications, to the field of dentistry, including in implantology [39,40]. Zirconia is available in both SM and AM [2]. The compressive strength of zirconia-based ceramics has been reported to be high, with a value of approximately 2000 MPa; the flexural strength ranges from 177 to 1000 MPa; the fracture toughness ranges from 1 to 8 MPa$\sqrt{m}$; the tensile strength ranges from 115 to 711 MPa; the hardness ranges from 5 to 15 GPa; and the modulus of elasticity ranges from 100 to 250 GPa [40]. Yttria-stabilized tetragonal zirconia polycrystal (Y-TZP) is currently the main material used for the application of zirconia-based ceramics to implant-supported restoration. Y-TZP is characterized by a well-known toughening mechanism attributed to the phase transformation of tetragonal to monoclinic phase [41].

Initially, Y-TZP was used as a substitute for the metal framework employed in metal–ceramic crowns and fixed dental prostheses. However, the veneering porcelain frequently experienced chipping due to interfacial binding problems between zirconia and the veneering ceramics [42,43]. Hence, monolithic Y-TZP restorations have recently been introduced for clinical trials, as the layering procedure can be omitted in such restorations for simplification of the digital processes adapting SM and AM [41].

With respect to the marginal accuracy, implant-supported monolithic Y-TZP fixed dental prostheses have been evaluated and shown to be clinically acceptable when prostheses are made by SM [44,45,46]. The accuracy ranges from 50 to 120 μm, although some previous studies have shown that the mean discrepancy value is less than 10 μm [44,46,47]. The clinically acceptable marginal gap is known to be less than 120 μm, which has been obtained from a previous clinical study of tooth-supported restorations [48]. Many authors have shown similar or more accurate results for SM-based restorations compared to those of fixed dental prostheses made by conventional methods [44]. However, many other articles have reported mean marginal gaps of less than 50 μm for cast gold restorations, all of which were published before 2010 [49,50,51]. Since 2011, many publications have indicated the excellent accuracy of SM-based dental prostheses. Measuring tools and skills have improved considerably, and marginal gaps have thus been measured more precisely. Notwithstanding, investigations on the marginal fit of SM-based restorations appear to have not only a bias in the selection of conventional materials, but also a tendency to show superiority in modern development [52,53,54,55,56].

There have been few papers reporting the long-term clinical results of fixed implant-supported monolithic Y-TZP restorations for periods of over five years [57,58,59,60,61,62,63,64]. The five-year survival rate is estimated to be over 90% in complete-arch fixed implant-supported monolithic Y-TZP prostheses [57,64]. There appear to be no studies on single or multiple-unit fixed implant-supported monolithic Y-TZP dental prostheses with a mean follow-up period of at least 3 years [62,63]. As SM-based monolithic Y-TZP restorations are being used in clinics globally, randomized controlled trials and other prospective clinical studies will be reported and published in the near future.

Some issues related to the dental use of monolithic Y-TZP still exist, including antagonistic dentition wear, the optimization of esthetics and mechanical properties, and low-temperature degradation after the exposure of Y-TZP to the oral environment. Monolithic Y-TZP with an adequate surface finish was reported to show an acceptable surface wear rate for the antagonistic dentition [65]. Minor elements for coloring and the esthetics of monolithic Y-TZP appear to have a limited influence on the mechanical properties [39,66]. The aging of Y-TZP through low-temperature degradation, representing a spontaneous transformation of the metastable tetragonal to monoclinic phase, has not been demonstrated to cause a problem in clinical performance [41,67]. Although none of these issues have been found to be clinically relevant, the issue of ceramic veneering on the Y-TZP framework is currently being addressed, especially because of its excellent esthetics in comparison with the monolithic form.

The five-year survival rates have recently been estimated to be 97.6% for Y-TZP-based ceramic implant-supported single crowns and 93.0% for multiple-unit fixed dental prostheses [62,63]. Complete-arch implant-supported fixed prostheses using veneered Y-TZP have a 97.7% five-year survival estimate [68]. However, the survival estimates of implant-supported restorations without any complications are much lower. Complication-free five-year survival rates have been revealed to be 83.8% for single crowns, approximately 70% for multiple-unit restorations, and approximately 60% for complete-arch fixed dental prostheses [42,62,63,68]. One of the major complications is the chipping of veneering porcelain, which is caused by various factors, including the supporting design of the Y-TZP framework, the mechanical properties of the veneering porcelain, and thermal residual stress at the interface due to differences in the coefficients of thermal expansion between Y-TZP and the veneering porcelain [41,42,43,69,70].

To reduce the number of porcelain chipping events, veneering ceramics are heated and pressed onto the Y-TZP framework to produce the final prostheses. SM technology is applied to Y-TZP frameworks, and heat-pressing using the traditional lost-wax technique is used for ceramic veneering on the framework. The veneering materials are glass ceramics, mainly lithium disilicate glass. Some mechanical test results of prostheses treated by this method have been reported [71,72,73]. However, clinical results are very rare, and few results can be found for implant-supported restoration [74]. Interestingly, a previous three-year randomized controlled clinical trial evaluating the survival rate of Y-TZP fixed dental prostheses with pressed veneering ceramics implied a tendency for more frequent chipping events, whereas a previous systematic review suggested superior clinical outcomes for the pressed technique [75,76].

Another approach to strengthening the bond between Y-TZP and the veneering porcelain is to use a resin cement. The Y-TZP framework and the veneering layer on the framework are made by SM, and the veneering layer is cemented to the framework [77]. A previous study reported that such a cementation technique is more resistant to interfacial failure in comparison to the manual layering technique [78]. However, the fracture resistance of frameworks produced by the cementation of Y-TZP and veneering materials is lower than that produced by manual layering and heat-pressing [78,79]. Therefore, many investigations on utilizing the fusion of glass ceramics to bond the lithium disilicate glass layer to the digitally machined Y-TZP have recently been conducted [41,77,80,81,82]. This technique of using the fusion of glass ceramics, so-called CAD-on, increases the resistance to adhesive failure between Y-TZP and lithium disilicate [82,83,84,85,86]. The marginal fit was shown to be acceptable (approximately 50 μm) for a single restoration made by CAD-on in a previous study [87]. One randomized controlled trial for tooth-supported 3-unit posterior fixed dental prostheses reported that this CAD-on technique was successful, resulting in less severe chipping events, although the mean follow-up period was approximately one year [88]. There have been no clinical investigations on implant-supported fixed CAD-on restoration, which are necessary.

Soft tissue responses to zirconia have been tested in depth. There are some investigations showing the cytotoxic response to Y-TZP of fibroblasts, which are the major cells of connective tissue [39]. However, recent studies, both in vitro and in vivo, have indicated favorable results for soft tissue integration into Y-TZP [39,89,90]. Partially stabilized zirconia materials, including Y-TZP, do not induce inflammatory or immune reactions when these materials are investigated in vitro and in vivo [39,91,92]. Regarding osseointegration into the zirconia surface, partially stabilized zirconia is biocompatible with bone tissues, although more numerous multinucleated giant cells and less bone-to-implant contact were found for the zirconia implants in a previous study, compared to the Ti implants [93,94]. Zirconia has been concluded to be a biocompatible material for implant-supported restoration.

As mentioned above, the application of AM technologies to the production of implant-supported ceramic restoration is still under way. Zirconia is an actively utilized and tested material for fabricating implant-supported ceramic prostheses [2]. The majority of 3D printers available in this field utilize stereolithography (SLA) [2]. Digital light projection (DLP) is one of the light sources that SLA uses to solidify photosensitive resin-coated ceramic suspensions contained in a vat [2]. The amount and characteristics of the binder (photosensitive resin), size of ceramic particles, sintering temperature, and sintering time are known to influence the surface quality, dimensional accuracy, and mechanical properties of the products of this AM process [8]. The surface roughness of the AM-based ceramics needs to be improved for implant-supported restoration. Although there have been no studies evaluating the dimensional accuracy of implant-supported zirconia restorations based on AM, SLA-manufactured ceramic crowns appear to have acceptable accuracy for clinical use [2,95]. It should be emphasized that further investigations and improvements are required for the realization of SLA-processed implant-supported zirconia restorations.

Besides the partially sintered Y-TZP used for SM and AM, fully sintered machinable (Y, Nb)-TZP may be employed to realize single-visit restorations. The trueness of the inner surface of crowns ground from the (Y, Nb)-TZP was found to be within the clinically acceptable range, with moderate bur wear [96]. Machinable (Y, Nb)-TZP is a modified form of tetragonal zirconia solid solution, containing both Y_2_O_3_ and Nb_2_O_5_, and has been utilized as the matrix phase in zirconia/alumina composites for implant abutments [97,98]. Machinability is defined as the relative ease or difficulty of removing material when transforming a raw material into a finished product. The machining of ceramics is conducted using a chip-forming process, so a high machinability involves cutting that should be restricted to the vicinity of the cutting tool. As the ease of chipping requires low hardness and high fracture toughness values for the localization of cutting, the composite quantity of toughness divided by hardness may represent the machinability [99]. The physical properties of (Y, Nb)-TZP and 3Y-TZP presented in Table 1 demonstrate the probable high machinability of (Y, Nb)-TZP compared to 3Y-TZP.

### 3.2. Lithium Disilicate Glass

Lithium disilicate (LS2) glass ceramic is one of the most popular all-ceramic systems currently used in the fabrication of single and multi-unit dental restorations due to its excellent mechanical and optical properties. Initially, this material was made commercially available as ingots, introduced to the market in 1998 with the name “IPS Empress 2” (Ivoclar Vivadent, Schaan, Liechtenstein), to be utilized according to the “heat-pressing” fabrication procedure, which is similar to the classic “lost wax” technique [100,101]. In 2005, a new formulation of LS2 was marketed as “IPS e.max Press” (Ivoclar Vivadent, Schaan, Liechtenstein), exhibiting improved mechanical properties and optical features which were much better than those of the older glass ceramics [101]. Recently, the chairside CAD/CAM systems have been recognized as reliable systems allowing the introduction of ceramic blocks aimed at the production of restorations by SM, and are also suitable for the chairside production of restorations [4,102,103,104]. LS2 is available for SM in digital dentistry, while it is unavailable for AM. In order to adapt the material to the needs of chairside CAD/CAM production processes, another technique has been introduced, which is based on the use of partially pre-crystallized blocks (IPS e.max CAD, Ivoclar Vivadent), containing both 40% lithium metasilicate (Li_2_SiO_3_) crystals and LS2 (Li_2_Si_2_O_5_) crystal nuclei; it is available in different shades and degrees of translucency, depending on the size and density of crystals [105]. This CAD/CAM material was initially available as a substructure material that afforded a greater translucency than other high-strength ceramic core materials. However, the CAD/CAM block has been used more recently for full-contour restorations of a single all-ceramic material (monolithic restorations) [106]. The IPS e.max CAD’s characteristics of high strength, the ability to be milled to full-contour esthetics, and dual placement (bond or cement) are useful in creating in-office implant restorations and thin veneers, or in any other situation in which strength and esthetics should be carefully balanced [5]. From in vitro studies, there is strong evidence that monolithic restorations, in contrast to bi-layered restorations, show fracture strength and fatigue resistance suitable for use in posterior areas, both in single implant-supported and 3-unit fixed prostheses [107,108,109,110,111]. In vitro fully anatomical e.max CAD crowns have been shown to exhibit fracture resistance that is suitable for posterior, monolithic restorations and to be more resistant to fatigue in cyclic loading than veneered zirconia, which is more prone to chipping [112,113]. LS2 glass ceramics have a relatively high flexural strength and fracture toughness for prosthodontic restorations. The flexural strength has recently been reported to range from 210 to 360 MPa, and the fracture toughness has been shown to range from 1.4 to 2.8 MPa$\sqrt{m}$ [114,115,116] (Table 2).

The marginal and internal fits of SM restorations depend on the precision of extraoral or intraoral digitalization, computer software design, milling, materials, and final sintering shrinkage [117,118]. Many studies have shown that LS2 restorations fabricated with the press technique have measurably more accurate marginal adaptations when compared to those fabricated with SM techniques [119]. However, the marginal fit of SM-based implant-supported LS2 restorations has been evaluated and demonstrated to be within the clinical acceptance level of 120 μm [44,48,55]. The restoration–manufacturing system and cementation procedure significantly influence marginal discrepancies, while materials have minor effects on the restoration accuracy [47]. The crystallization process of e.max CAD results in 0.3% shrinkage, affecting the marginal adaptation of the definitive restoration with no significance [120].

Fatigue behaviors are important for the clinical long-term prognosis of prosthodontic restorative materials. The results of several studies have shown that SM-based monolithic LS2 implant-supported single crowns survived after dynamic loading, thermocycling, and aging [121,122,123,124]. The full anatomical e.max CAD crowns prepared in monolithic form provide fatigue resistance by increasing the mechanical stability, which is enabled by the ability of a single-piece structure to withstand higher forces. One of these studies evaluated different CAD/CAM materials for monolithic implant-supported molar crowns [123]. After thermal and mechanical fatigue, the LS2 group was the most resistant, compared to the resin-modified and feldspathic groups. The LS2 group showed the highest flexural strength and modulus of elasticity. LS2 had a fracture resistance which was even higher than that of zirconia-reinforced lithium silicate (ZLS). However, ZLS revealed superior mechanical properties (fracture toughness, flexural strength, elastic modulus, and hardness) to LS2 glass ceramics in another study that only evaluated the mechanical properties [125].

There are many studies reporting clinical results related to implant prostheses of LS2. However, there is a lack of long-term clinical studies on LS2 restorations made by SM. A previous clinical study showed the two-year prospective results obtained from single implant-supported monolithic LS2 crowns fabricated by complete digital workflow, reporting that the implant LS2 restorations in premolar and molar sites had survival rates of 100%, without any technical or biological complications after two years [126]. A three-year cross-sectional retrospective study indicated that monolithic LS2 restorations were reliable and suitable clinical options for restoring a posterior missing tooth on a dental implant, considering that 89% of the participants in the LS2 group were free of complications [36]. Only one participant experienced minor chipping, affecting the LS2 restoration. A five-year prospective cohort study showed promising results, including 100% survival with no failures in SM-based single implant-supported LS2 crowns [127]. Implant-supported LS2 prostheses have technical complications, such as minor chipping and occlusal roughness. The increased roughness of LS2 ceramics over time in the oral cavity is presumably the result of numerous factors, including tooth brushing, environmental conditions, abrasion, attrition by antagonistic wear, and grinding damage [128,129,130,131,132,133]. Surface roughness may be strongly related to bacterial adherence and fracture resistance [134,135,136].

With regard to soft tissue reactions, LS2 materials do not appear to be any more cytotoxic than other dental restoration materials; they have been demonstrated to be less cytotoxic than several commonly used composite materials and have cytotoxicity comparable to that reported for several alloys and glass ionomers [137,138,139]. Furthermore, the fabrication methods (analog vs. digital) have no significant effect on the basic biological behavior. As a result, the LS2s are not biologically inert, and many have a similar cytotoxicity dynamic regardless of small differences in the composition or processing [140]. However, surface treatments affect the biological response. Polished LS2 ceramics exhibited improved adhesion and proliferation, compared to glazed LS2 ceramics [141]. The abrasion provides microrough surfaces with a strong wettability, allowing the strong adhesion of epithelial and connective tissues, which is advantageous in the soft tissue seal [142]. By contrast, applying the glazing technique to the same material will provide a smooth surface with strong hydrophobicity, which gives the material restricted adhesion properties, and this is appropriate for a dental surface designed to prevent biofilm formation in the septic environment of the mouth [143].

The texture of a ceramic surface affects the wear of opposing materials and mechanical properties. In recent studies, the wear resistance and abrasiveness of CAD/CAM materials have been evaluated [144]. In this in vitro study, the nanofilled composite resin and polymer-infiltrated ceramic were more antagonist-friendly (whether enamel or CAD/CAM materials) than glass ceramics and zirconia. However, another in vivo study reported that glass matrix materials showed less wear on the opposing enamel surface than resin matrix ceramic materials [145]. The most abrasive materials for antagonist enamel were the resin matrix ceramic groups, and not the glass matrix ceramic groups, probably because of the earlier abrasion of the polymer parts of these materials; their ceramic components did not exhibit this abrasion. In addition, the mechanical strength of the glazed ceramic was lower than that of the polished ceramic due to the amorphous and porous ceramic surfaces. Taken together, the use of a polished surface, rather than a glazed one, is recommended for implant-supported prostheses made of LS2.

## 4. Conclusions

A digital workflow has been established for dental restoration and includes data acquisition, data processing, and data manufacturing. Due to esthetics and durability, dental ceramics have been used as a material of choice for implant-supported restoration. Two categories of ceramic materials are widely used in clinics and are being advanced for the application of data manufacturing.

Y-TZP, belonging to zirconia-based ceramics, has been utilized globally in fabricating implant-supported prostheses. This material can be monolithic or a core material with veneering. Y-TZP is available for both SM and AM, which is still under development for prosthodontic application. LS2, belonging to glass ceramics, is another globally used material for implant-supported prostheses. LS2 is inferior to Y-TZP in terms of its mechanical properties, while it is superior in terms of its esthetics. This material is only available for SM.

There has been a lack of long-term clinical studies for implant-supported restorations made of Y-TZP and LS2. Further clinical evaluations of these materials are required. Additionally, the improvement of ceramic materials is necessary for dental application, especially implant prosthodontics. The material selection of a dental clinician remains an important issue because more esthetic materials are usually less durable in dental ceramics than stronger ones.

## Figures and Tables

**Table 1:** Comparison of the mechanical properties of (Y, Nb)-tetragonal zirconia polycrystal (TZP) and 3Y-TZP.

Mechanical Properties	(Y, Nb)-TZP	3Y-TZP
Biaxial strength, MPa	760	1010
Fracture toughness, MPam^1/2^	7.4	6.0
Hardness, GPa	8.5	13.2
Modulus of elasticity, GPa	174	220
Transmittance *, Ratio	1.1	1

* Relative transmittance when 3Y-TZP transmittance is calibrated as 1.

**Table 2:** Comparison of the mechanical properties of lithium disilicate glass (LS2) and 3Y-TZP.

Mechanical Properties	LS2	3Y-TZP
Biaxial strength, MPa	360	1010
Fracture toughness, MPam^1/2^	2.5	6.0
Hardness, GPa	5.8	13.2
Modulus of elasticity, GPa	95	220
Thermal expansion coefficient *, 10^−6^/K	10.5	10.5
Transmittance **, %	1.1–1.4	1

* Temperature range: 100–500 °C. ** Relative transmittance when 3Y-TZP transmittance is calibrated as 1.
